# Network Analysis of Sleep Quality and Psychiatric Symptoms Among ICU Nursing Staff

**DOI:** 10.1155/jonm/1469015

**Published:** 2026-04-27

**Authors:** Yating Li, Yan Zhang, Wenjin Chen, Wei He, Jie Jian, Jingyi Xu, Yang Sun, Xiaoguo Ma, Ziyi Ding, Di Zhao, Haishui Shi

**Affiliations:** ^1^ Hebei Key Laboratory of Early Life Health Promotion (SZX202419), Hebei Medical University, Shijiazhuang, China, hebmu.edu.cn; ^2^ School of Nursing, Hebei Medical University, Shijiazhuang, China, hebmu.edu.cn; ^3^ Handan Central Hospital, Handan, China, hdzxyy.com; ^4^ Department of Neurosurgery, Xuanwu Hospital, Capital Medical University, Fengtai, Beijing, China, ccmu.edu.cn; ^5^ Department of Critical Care Medicine, Beijing Tongren Hospital, Capital Medical University, Fengtai, Beijing, China, ccmu.edu.cn; ^6^ Department of Neurosurgery, The Fourth Hospital of Hebei Medical University, Shijiazhuang, China, hebmu.edu.cn; ^7^ The Key Laboratory of Neural and Vascular Biology, Ministry of Education, Hebei Medical University, Shijiazhuang, China, hebmu.edu.cn

**Keywords:** intensive care unit, network analysis, nurses, psychiatric symptoms, sleep quality

## Abstract

**Background:**

Intensive care unit (ICU) nurses are at high risk for sleep problems and psychological symptoms. This study aimed to construct a network model to explore the interrelationships between sleep quality and psychiatric symptoms among ICU nurses and to identify central and bridge symptoms for precise intervention.

**Methods:**

A multicenter cross‐sectional study was conducted from January to March 2025 among registered nurses working in ICUs. Psychiatric symptoms were assessed using the Symptom Checklist‐90 (SCL‐90), and sleep quality was measured using the Pittsburgh Sleep Quality Index (PSQI). A Gaussian Graphical Model was estimated using the EBICglasso algorithm. Centrality indices (strength, closeness, betweenness, and expected influence) and bridge centrality were calculated to identify key symptoms. The stability of the network was assessed using nonparametric and case‐dropping bootstrap analyses.

**Results:**

A total of 5560 nurses were included in the analysis. The network model revealed a well‐connected structure. Centrality analysis indicated that “subjective sleep quality”, “anxiety”, and “sleep and eating problems” were the most central symptoms in the entire network. Bridge centrality analysis identified “sleep and eating problems” as the most critical bridge symptoms, forming the strongest connections between the sleep and psychiatric symptom communities. The network demonstrated excellent stability, with a correlation stability coefficient of 0.75 for both strength and bridge strength.

**Conclusion:**

The findings highlight the pivotal roles of subjective sleep quality, sleep and eating problems, and anxiety as potential targets for precise interventions. Focusing on these symptoms may effectively disrupt the vicious cycle between poor sleep and psychological distress, thereby improving overall well‐being.

## 1. Introduction

Nurses working in the intensive care unit (ICU) operate in a highly demanding and stressful environment, which has led to growing concern regarding their physical and mental well‐being [1]. Substantial evidence indicates that ICU nurses are a high‐risk group for both sleep problems and psychiatric symptoms [2]. The inherent characteristics of their profession, such as shift work, managing critical patients, and frequent exposure to death, predispose them to poor sleep quality and a range of mental health issues [3, 4]. Compounding this problem is the bidirectional relationship between sleep disturbances and psychiatric symptoms, where each can act as a risk factor for the other, potentially creating a vicious cycle that severely impacts nurses’ job performance, burnout, and overall quality of life [5].

Conventional research often treats sleep and psychiatric problems as distinct entities or focuses solely on their comorbidity, falling short of elucidating the intricate interplay among individual symptoms [6]. The network theory of mental disorders, proposed by Borsboom, offers a novel framework for addressing this limitation. As a practical application of this theory, network analysis is a graph‐theoretic statistical approach that visually represents this complex system using “nodes” (symptoms) and “edges” (associations between symptoms) [7]. It allows for the identification of central symptoms (highly influential within a disorder) and bridge symptoms (crucial for connecting two distinct disorders), which may act as drivers of the entire psychopathological process [8].

Although network analysis has been applied in various populations, few have specifically modeled the symptom‐level interplay between sleep and psychiatric symptoms among ICU nurses in China. Therefore, this study aims to employ network analysis to construct a network model depicting the relationships between sleep quality and psychiatric symptoms among Chinese ICU nursing staff. Through this data‐driven exploration, we seek to identify key bridge symptoms that connect sleep problems with psychological issues, thereby revealing the underlying mechanisms [9]. The findings are expected to provide practical guidance for developing precise interventions that focus on these pivotal symptom targets.

## 2. Materials and Methods

### 2.1. Study Design and Participants

This multicenter cross‐sectional study was conducted from January to March 2025 among registered nurses working in ICUs across approximately 300 cities in China using convenience sampling. Inclusion criteria were current employment as a clinical nurse in an ICU and possession of a valid registered nurse license. Nurses on leave and nursing students were excluded. Before data collection, all participants were assured of the anonymity and confidentiality of their responses. The study protocol was approved by the Ethics Committee of Hebei Medical University (Approval No.: 20,190,436). The study was reported in accordance with the Strengthening the Reporting of Observational Studies in Epidemiology Statement.

### 2.2. Measures

Sociodemographic characteristics were collected using a self‐designed questionnaire to collect information on age, gender, marital status, education level, length of employment, professional title, hospital level, night shift frequency, and health‐related characteristics. Age was categorized into four groups including 20–29, 30–39, 40–49, and ≥ 50 years. Night shift frequency was categorized into three levels including ≤ 3 days per night shift, > 3 days per night shift, and no night shifts. Previous studies have shown that age, gender, and night shift frequency are associated with sleep quality and psychiatric symptoms, and these variables were treated as covariates [10, 11].

The psychiatric symptoms of ICU nurses were assessed using the Chinese version of the Symptom Checklist‐90 (SCL‐90). The SCL‐90 is a 90‐item self‐report instrument that measures psychopathological symptoms across 10 factors: somatization, obsessive‐compulsive symptoms, interpersonal sensitivity, depression, anxiety, hostility, phobic anxiety, paranoid ideation, psychoticism, and an additional domain that reflects sleep and eating problems. Each item is rated on a five‐point Likert scale, from 1 (not at all) to five (extremely). The SCL‐90 factor scores were calculated as the mean of the items contributing to each factor. The total SCL‐90 score was calculated by summing the scores of all 90 items, with a score ≥ 160 considered indicative of positive screening for psychiatric symptoms [12].

Sleep quality was assessed using the Pittsburgh Sleep Quality Index (PSQI), a 19‐item self‐rated questionnaire that evaluates seven components: subjective sleep quality, sleep latency, sleep duration, habitual sleep efficiency, sleep disturbances, use of sleeping medication, and daytime dysfunction. The Chinese version of the PSQI has shown good psychometric properties. The total PSQI score ranges from 0 to 21, with higher scores indicating poorer sleep quality, and a score > 7 is commonly used to identify poor sleep quality [13].

### 2.3. Sample Size

The sample size for network analysis was determined based on the number of estimated parameters. For a network with *N* nodes, the number of parameters includes *N* threshold parameters and *N* (*N-*1)/2 pairwise association parameters [14], yielding a total of *N* + *N* (*N-*1)/2. In the present study, the network comprised 20 nodes, including 10 psychiatric symptom dimensions from the SCL‐90, 7 sleep quality components from the PSQI, and 3 covariates (age, gender, and night shift frequency). Thus, the total number of estimated parameters was 210. To ensure stable estimation and reliable centrality indices, methodological recommendations suggest a ratio of 3–5 participants per estimated parameter [15]. Based on this criterion, the required sample size for the present network ranged from 630 to 1050 participants. The final sample of 5560 ICU nurses exceeded the required sample size, ensuring that the identified network structure, central symptoms, and bridge symptoms are highly reliable.

### 2.4. Statistical Analyses

Descriptive statistical analyses were performed using SPSS software. Continuous variables are presented as mean and standard deviation or median and interquartile range. Count data are expressed as numbers and percentages.

Network models were constructed using *R* software. A network was estimated using the EBICglasso method. To account for potential confounding effects, the study included age, gender, and night shift frequency as additional nodes in the network.

Centrality analysis was performed to identify the most influential nodes within the network using four indices: strength, closeness, betweenness, and expected influence. Strength was defined as the sum of the absolute values of the edge weights between a node and all nodes directly connected to it. Closeness was defined as the inverse of the sum of the shortest path distances from a node to all other nodes in the network. Betweenness referred to the number of times a node appeared on the shortest paths between any two nodes in the network. Expected influence reflects a node’s net impact on the network by considering both the magnitude and direction of its associations. Bridge centrality measures the extent to which a node serves as a connector between different communities within a network. Bridge strength and bridge expected influence were calculated to identify symptoms that act as crucial connectors between the predefined communities [16].

To ensure the robustness of the network analysis, we evaluated network accuracy and stability with edge weight accuracy assessment and centrality stability analysis. Edge accuracy was assessed using 95% nonparametric confidence intervals (CIs) derived from 500 bootstrap samples. Narrower CIs were indicative of more precise and stable edge weight estimates. Stability of the network was assessed using the case‐dropping bootstrap (250 samples). The correlation stability coefficient (CS‐coefficient) was calculated; a value above 0.25 indicates moderate stability, and above 0.5 indicates strong stability [17].

## 3. Results

### 3.1. Participant Characteristics

The sociodemographic characteristics of the 5560 participants are presented in Table 1. The majority were female (85.83%), married (67.16%), and held a bachelor’s degree (83.70%). The largest age group was 30–39 years (52.72%). Most participants worked in tertiary hospitals (77.36%).

**TABLE 1:** Characteristics of ICU nurses.

**Item**		***n***	**%**

Gender	Male	788	14.17
	Female	4772	85.83

Age	20–29	1983	35.67
	30–39	2931	52.72
	40–49	589	10.59
	≥ 50	57	1.03

Marital status	Married	3734	67.16
	Unmarried/divorced	1826	32.84

Education	Master’s degree and above	78	1.40
	Bachelor	4654	83.70
	Junior college	828	14.90

Diabetes	Yes	100	1.80
	No	5460	98.20

Hypertension	Yes	190	3.42
	No	5370	96.58

Smoking	Yes	342	6.15
	No	5218	93.85

Drinking	Yes	3361	60.45
	No	2199	39.55

BMI	< 18.5	684	12.30
	18.5–23.9	3254	58.53
	24–27.9	1177	21.17
	> 28	445	8.00

Length of employment	0–5 years	1521	27.36
	6–10 years	1637	29.44
	11–15 years	1527	27.46
	> 15 years	875	15.74

Night shift frequency	≤ 3 days per night shift	1576	28.35
	> 3 days per night shift	3725	67.00
	No night shifts	259	4.65

Professional title	Junior	2950	53.06
	Mid‐level	2245	40.38
	Associate Senior	323	5.81
	Senior	42	0.76

Hospital level	Tertiary hospital	4301	77.36
	Secondary hospital	1259	22.64

ICU night shift nurse‐to‐bed ratio	1:1	312	5.61
	1:2	1212	21.80
	1:3	1907	34.30
	1:4	1113	20.02
	1:4+	1016	18.27

### 3.2. Psychiatric Symptoms and Sleep Quality

The scores for the SCL‐90 and PSQI are shown in Tables 2 and 3, respectively. The total SCL‐90 score was 172.07 ± 73.74, and 45.70% of the nurses scored ≥ 160, suggesting a risk for psychological symptoms. The total PSQI score was 7.69 ± 3.67, with 46.76% of the nurses scoring > 7, indicating poor sleep quality.

**TABLE 2:** Psychiatric symptoms of ICU nurses.

	**Mean**	**SD**

Somatization	1.98	0.85
Obsessive‐compulsive	2.25	0.91
Interpersonal sensitivity	1.89	0.88
Depression	2.02	0.91
Anxiety	1.86	0.87
Hostility	1.90	0.89
Photic anxiety	1.61	0.82
Paranoid ideation	1.76	0.85
Psychoticism	1.71	0.84
Sleep and eating problems	1.95	0.88
Total score	172.07	73.74

**TABLE 3:** Sleep quality of ICU nurses.

	**Mean**	**SD**

Subjective sleep quality	1.37	0.82
Sleep latency	1.65	0.93
Sleep duration	1.39	0.90
Sleep efficiency	0.52	0.87
Sleep disturbances	1.29	0.68
Use of sleeping medication	0.30	0.73
Daytime dysfunction	1.21	1.04
Total score	7.69	3.67

### 3.3. Network Structure and Centrality

**FIGURE 1:**
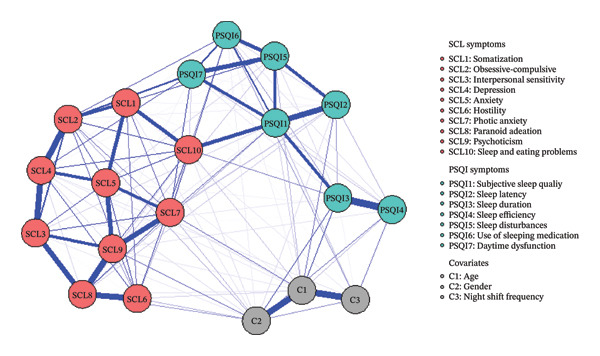
Network model of sleep quality and psychiatric symptoms.

The network structure comprised 10 nodes from the SCL‐90, 7 nodes from the PSQI, and 3 covariates. The estimated network had a density of 0.63. This network is visualized in Figure 1. The layout clearly delineates the psychiatric symptoms community (red nodes), the sleep quality community (green nodes), and the covariates (gray nodes). The thickness of lines corresponds to the strength of the association.

**FIGURE 2:**
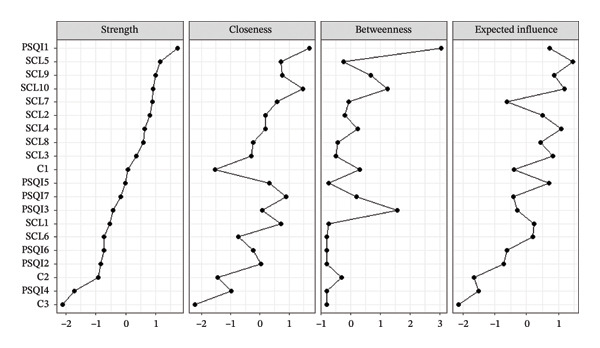
Centrality plot of the network (strength, closeness, betweenness, and expected influence).

The centrality indices of strength, closeness, betweenness, and expected influence were used to identify the most influential symptoms within the entire network (Figure 2). Strength centrality identified PSQI1 (subjective sleep quality) has the highest strength, followed by SCL5 (anxiety), indicating they are the most connected nodes in the network. Closeness centrality indicates PSQI1 (subjective sleep quality) and SCL10 (sleep and eating problems) rank highest in Closeness. PSQI1 (subjective sleep quality) as having the highest betweenness, meaning it acts as a critical bridge between other nodes in the network. In addition, SCL5 (anxiety) and SCL10 (sleep and eating problems) emerge as the most influential nodes, with the highest expected influence.

To identify symptoms that are crucial for connecting the sleep and psychiatric symptom clusters, bridge centrality was computed. Bridge centrality analysis further highlighted SCL10 (sleep and eating problems) as the most critical bridge symptom, forming the strongest connections between the communities (Figure 3).

FIGURE 3Plot of bridge strength centrality (z‐scores).

**Figure:**
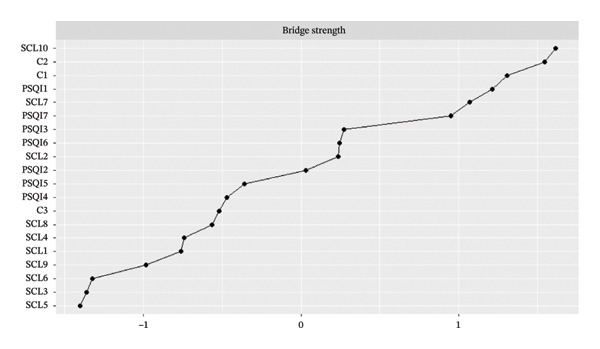
(a)

**Figure:**
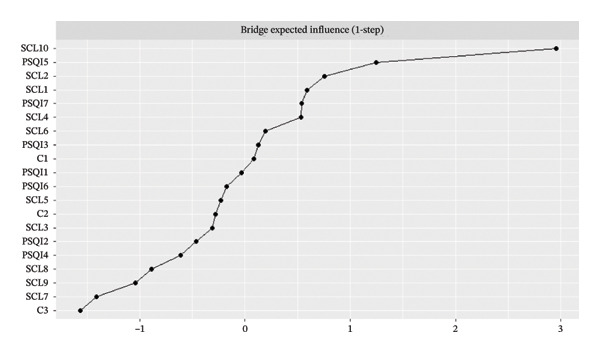
(b)

### 3.4. Network Accuracy and Stability

The robustness of the network estimation was rigorously tested. The non‐parametric bootstrap (500 samples) indicated good accuracy for edge weights, with generally narrow 95% CIs for most edges (Figure 4(a)). Furthermore, the case‐dropping bootstrap (250 samples) was employed to assess the stability of the centrality indices. The results demonstrated excellent stability for the network (Figure 4(b)). The CS‐coefficient was 0.75 for both strength and bridge strength. This value substantially exceeds the recommended threshold of 0.50, indicating that the identified network structure and the centrality of key symptoms are highly reliable and not influenced by random variations in the sample.

FIGURE 4Accuracy and stability analysis of the network. (a) Accuracy analysis of the edge weights. (b) Stability analysis of the centrality indices.

**Figure:**
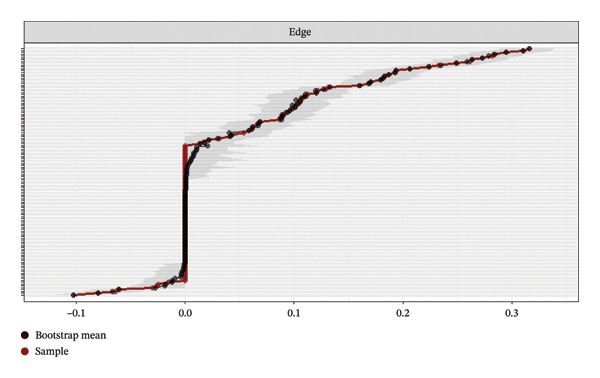
(a)

**Figure:**
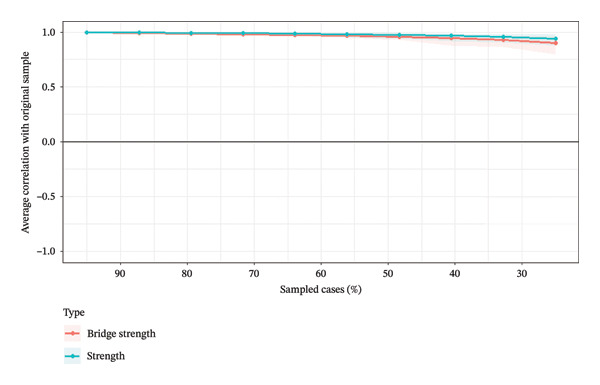
(b)

## 4. Discussion

This study employed network analysis to elucidate the intricate relationships between sleep quality and psychiatric symptoms among a large cohort of 5560 ICU nurses in China. The main findings reveal that “subjective sleep quality” (from PSQI1), “anxiety” (from SCL‐90), and “sleep and eating problems” (from SCL‐90) are the most central symptoms within the network. Moreover, “sleep and eating problems” serve as the most influential bridge symptoms, linking the sleep‐related and psychiatric symptom clusters. Their high centrality and bridge indicate that they are densely connected to many other nodes, suggesting that these symptoms may act as key drivers in the activation and maintenance of the overall pathological state [18].

The high centrality of “subjective sleep quality” is profound. It suggests that an ICU nurse’s personal appraisal of their sleep is a component that strongly influences, and is influenced by, a wide array of other psychological and somatic symptoms. This node appears to be a core driver of overall well‐being [19].

The centrality of “anxiety” aligns with a vast body of research highlighting anxiety symptomatology as a cornerstone of mental health crises in high‐stress professions, including nursing [20]. Its position as a highly connected hub in the network suggests it functions as a symptom amplifier, potentially exacerbating other issues like depression, photic anxiety, psychoticism, and somatization. This finding is consistent with other studies that have identified anxiety symptoms as core to their psychological distress [21, 22].

The critical finding is that “sleep and eating problems” are the most significant bridge symptoms. This bridge can be mechanistically explained through shared neurobiological pathways [23]. Chronic stress and shift work, inherent to ICU nursing, disrupt the hypothalamic‐pituitary‐adrenal axis and circadian rhythms [24, 25]. This dysregulation impairs both sleep architecture and the systems that govern appetite and emotional control [26]. The stress hormones (e.g., cortisol) that fragment sleep also influence appetite‐regulating peptides (e.g., leptin and ghrelin), leading to eating problems [27, 28]. Therefore, the “sleep and eating problems” node is not merely a symptom but a tangible manifestation of maladaptive stress response. It serves as a bridge because it is a common outcome of psychological distress and a direct cause of poor sleep, thereby creating a bridge between the two symptom communities [29].

The identification of central and bridge symptoms offers a strategic, mechanism‐based target for interventions. For instance, Cognitive Behavioral Therapy for Insomnia, which specifically targets the catastrophic beliefs about sleep underlying “subjective sleep quality,” could be particularly effective. Concurrently, interventions focusing on emotional regulation and stress management (e.g., mindfulness) could help stabilize the hypothalamic‐pituitary‐adrenal axis, thereby improving sleep quality and normalizing eating patterns [30, 31], effectively dismantling the “sleep and eating problems” bridge [32].

While some studies have established a correlational link between poor sleep and mental health in healthcare workers [33], our study advances the field by adopting the network approach, which conceptualizes disorders not as latent entities but as systems of interacting symptoms. In addition, the methodological rigor of our study is also a key differentiator. The large sample size provides substantial statistical power. Furthermore, the network was estimated using the state‐of‐the‐art EBICglasso algorithm, a method known for producing sparse, interpretable, and replicable networks. The stability of the network was rigorously tested using bootstrapping techniques, a best‐practice standard for ensuring the reliability of network metrics. The resulting high CS‐coefficient of 0.75 for both strength and bridge strength provides strong confidence that the identified central and bridge symptoms are robust.

However, several limitations must be acknowledged. First, the study’s cross‐sectional design precludes any definitive conclusions about causality. Second, the data were collected via self‐report questionnaires. Although these are validated instruments, they are susceptible to reporting biases. Future studies could incorporate objective measures of sleep, such as actigraphy or data from wearable devices, to complement the subjective reports. Third, while this study included age, gender, and night shift frequency as covariates to account for potential confounding effects, other factors such as social support and job burnout, which may also be associated with sleep quality and psychiatric symptoms, should be considered in future research.

## 5. Conclusion

This network analysis provides a detailed understanding of the interactions between sleep and psychiatric symptoms among ICU nurses. The findings highlight the pivotal roles of subjective sleep quality, sleep and eating problems, and anxiety as strategic points of intervention. By focusing resources on improving nurses’ subjective sleep and mitigating the behavioral disruptions, healthcare organizations may be able to effectively break the vicious cycle, ultimately fostering a healthier nursing workforce.

## Author Contributions

Conception or design of the work: Haishui Shi, Di Zhao, and Yating Li. Data collection: Yan Zhang, Wenjin Chen, Wei He, Jie Jian, Jingyi Xu, Yang Sun, Xiaoguo Ma, and Ziyi Ding. Analysis or interpretation of data: Yating Li and Yan Zhang. Drafting the work: Yating Li and Haishui Shi.

## Funding

This work was partly supported by the 2025 Hebei Provincial Social Science Development Research Project (HBSKFZ25QN120), the National Natural Science Foundation of China (82171536), the Key laboratory of Neural and Vascular Biology, Ministry of Education of China (NV20250010), the Medical Science Research Project of Hebei (20250178), the Project from Handan Municipal Bureau of Science and Technology (23422083010ZC), and the Undergraduate Innovative Experimental Program of Hebei Medical University (USIP2024084).

## Conflicts of Interest

The authors declare no conflicts of interest.

## Data Availability

The data that support the findings of this study are available from the corresponding author upon reasonable request.
